# Cementation of Glass-Ceramic Posterior Restorations: A Systematic Review

**DOI:** 10.1155/2015/148954

**Published:** 2015-10-18

**Authors:** Carline R. G. van den Breemer, Marco M. M. Gresnigt, Marco S. Cune

**Affiliations:** ^1^University Medical Center Groningen, Center for Dentistry and Oral Hygiene, Department of Fixed and Removable Prosthodontics and Biomaterials, The University of Groningen, Groningen, Netherlands; ^2^St. Antonius Hospital Nieuwegein, Department of Oral-Maxillofacial Surgery, Prosthodontics and Special Dental Care, Nieuwegein, Netherlands

## Abstract

*Aim*. The aim of this comprehensive review is to systematically organize the current knowledge regarding the cementation of glass-ceramic materials and restorations, with an additional focus on the benefits of Immediate Dentin Sealing (IDS). *Materials and Methods*. An extensive literature search concerning the cementation of single-unit glass-ceramic posterior restorations was conducted in the databases of MEDLINE (Pubmed), CENTRAL (Cochrane Central Register of Controlled Trials), and EMBASE. To be considered for inclusion, *in vitro* and *in vivo* studies should compare different cementation regimes involving a “glass-ceramic/cement/human tooth” complex. *Results and Conclusions*. 88 studies were included in total. The *in vitro* data were organized according to the following topics: (micro)shear and (micro)tensile bond strength, fracture strength, and marginal gap and integrity. For *in vivo* studies survival and quality of survival were considered. *In vitro* studies showed that adhesive systems (3-step, etch-and-rinse) result in the best (micro)shear bond strength values compared to self-adhesive and self-etch systems when luting glass-ceramic substrates to human dentin. The highest fracture strength is obtained with adhesive cements in particular. No marked clinical preference for one specific procedure could be demonstrated on the basis of the reviewed literature. The possible merits of IDS are most convincingly illustrated by the favorable microtensile bond strengths. No clinical studies regarding IDS were found.

## 1. Introduction

Bonded glass-ceramic restorations have gained popularity, particularly after new materials, bonding systems, cements, and cementation techniques became available in recent years. Nowadays different ceramics are introduced for the use of posterior restorations, being either an oxide-ceramic or a glass-ceramic. Glass-ceramics are of special interest in this review because their silica content and micromechanical interlocking structure allow adhesive cementation to enamel and dentin. Consequently, glass-ceramic restorations can withstand tensile forces without cement failure, even if the preparation of the tooth is nonretentive. Since the surface treatment of feldspathic porcelain in 1983 [1] became available, new materials have evolved into high strength and esthetic glass-ceramics such as lithium disilicate. This higher strength compared to earlier glass-ceramics is reached because of a different firing process [2]. Contemporary glass-ceramic fixed dental crowns possess good optical and mechanical properties, thus mimicking natural teeth to a large extent [3–5].

To ensure proper attachment of an indirect restoration, basically two aspects have to be taken into consideration: conditioning of the ceramic material and conditioning of the tooth substrate followed by cementation. The most commonly used conditioning method for the glass-ceramic surface these days is application of hydrofluoric acid and silanisation, as reviewed by Tian et al. [6]. Cements are considered necessary to obtain durable retention of the restoration and good marginal seal, as well as maintaining original color and marginal outline. The first dental luting agents were water based cements like zinc phosphate and glass ionomer cements. With the introduction of resin cements, properties like solubility and adhesion improved, thereby allowing a minimally invasive preparation design [7]. Contemporary resin cements vary in properties like viscosity, whether or not they need light curing, and whether they are adhesive, self-etching, or self-adhesive. However these cements require some kind of conditioning procedure of the tooth substrate and indirect restoration.

In addition, sealing of dentin tubules with a filled adhesive resin directly after tooth preparation and prior to (digital or analogue) impression taking is presumed to result in improved bond strength, less gap formation, decreased bacterial leakage, and reduced dentin sensitivity [8]. This procedure may be highly clinically relevant and was first tested* in vitro* by Pashley et al. [9] and described in 1996 as the dual application of dentin bonding agents [10]. Later Magne referred to it as “Immediate Dentin Sealing” (IDS) [8].

Compared to luting with water based cements, adhesive cementation is more difficult and time-consuming and moisture control is more important. A clinical study showed a tendency to higher fracture rates among posterior crowns compared to anterior crowns, and indirect bonded restorations in molars revealed higher failure rates than premolar crowns [11]. Hence cementation of glass-ceramics in the posterior region appears clinically the most challenging and thus is of clinical relevance for further investigation. There is little homogeneity between studies in terms of materials, test method, and analysis. For* in vitro* studies four types of testing are predominantly applied: (micro)shear bond strength, (micro)tensile bond strength, fracture strength, and marginal gap. The outcomes of these studies are of importance as this could predict the long term results of indirect restorations.

A shear bond strength test evaluates the degree to which two attached specimens resist shear. A true shear test is difficult to perform because one of the specimens is always fixed to the test device. Instead, a microshear bond strength test is preferable, in which a cross-sectional area of 1 mm^2^ is generally used for greater uniformity of stress distribution. This test results in more adhesive failures at the bonding interface instead of cohesive failures in the substrate, which is considered to be more realistic [6].

A tensile bond strength test is performed perpendicular to the bonded interface and is therefore generally adopted as the most valid bond strength test at this moment [12]. However it is hard to control the alignment of specimen, and nonuniform stress distribution across the bonding surface occurs. With a microtensile test the small size of the specimen leads to a more favorable stress distribution and to bond failures that lie closer to their ultimate strengths [13].

Fracture loading, fracture resistance, load-to-failure, breaking strength, and fracture strength are considered synonymous terms. They are used to indicate the stress at which a specimen fails by occlusal loading, and, in the following, the term “fracture strength” will be adopted. In general, restored teeth are progressively, occlusally loaded until fracture by means of a stainless steel ball. Fracture strength and fracture type are the most common outcome parameters.

The marginal gap reflects the quality of marginal adaptation and is commonly studied by means of microleakage experiments (e.g., with dye penetration or silver staining and/or by scanning electron microscopy (SEM)), either with or without thermocycling and with or without loading in a chewing simulator. With conventional nonadhesive restorations the size of the marginal gap is considered of paramount importance for the (quality of) survival of the restoration and should be as small as possible. The size of the marginal gap may not be as critical when using materials that can be luted adhesively to the tooth substrate, such as glass-ceramics.

There appears to be a plethora of materials, cements, bonding systems, and cementation techniques for luting glass-ceramics to posterior teeth. The aim of this systematic review is to focus on cements and organize the current knowledge and the manner in which cements are used for the cementation of glass-ceramic materials and restorations, with an additional focus on the benefits of IDS.

## 2. Materials and Methods

### 2.1. Search Strategy

A comprehensive literature search was undertaken in the databases of MEDLINE (1950–1 January 2015) (Pubmed), CENTRAL (1800–1 January 2015) (Cochrane Central Register of Controlled Trials), and EMBASE (1966–1 January 2015) by means of a combination of MeSH terms and text words. The English language restriction was applied and articles without an available abstract were not considered. The search strategy is outlined as follows.

*Search Strategy*

*MEDLINE*. ((“Ceramics”[Mesh] OR ceramic∗[tw]) AND (“Cementation”[Mesh] OR “Dental Cements”[Mesh] OR cementation∗[tw] OR immediate dentin seal∗[tw] OR luting[tw] OR lute[tw] OR dental adhesives[tw] OR resin coat∗[tw])) NOT (veneer∗[TI] OR posts∗[TI] OR implant∗[TI] OR zirconi∗[TI] OR alumina[TI] OR “zirconium oxide”[Supplementary Concept]) NOT (“Case Reports”[Publication Type] OR “Review”[Publication type]) AND English[lang].

Run data search: January 1, 2015 (1868 results).

*EMBASE. *“dental ceramics”/exp OR ceramic∗:ab,ti AND (“cementation”/exp OR “tooth cement”/exp OR cementation∗:ab,ti OR “immediate dentin sealing”:ab,ti OR luting:ab,ti OR lute:ab,ti OR “dental adhesives”:ab,ti OR “resin coating”:ab,ti).

NOT (veneer∗:ti OR posts∗:ti OR implant∗:ti OR zirconi∗:ti OR alumin∗:ti) NOT (“case report”/exp OR “review”/exp) AND[english]/lim.

Run data search: January 1, 2015 (806 results).

*COCHRANE Library (Trials) (Search in ti,ab,kw)*. ceramic∗ AND (cement∗ OR immediate dentin seal∗ OR luting OR lute OR dental adhesive∗ OR resin coat∗).

Run data search: January 1, 2015 (332 results).

### 2.2. Study Selection

Titles and abstracts of the identified publications were screened by one of the authors. Full text documents were obtained for all articles meeting the inclusion criteria. Additional hand searching was performed by following up on the reference lists from included articles. Full text analysis to decide on inclusion/exclusion was subsequently performed by two reviewers and Cohen's Kappa was used as the measure of agreement. Disagreements were resolved by manner of discussion.

Methodological quality regarding the risk of bias in selected articles was assessed by one of the authors according to the criteria as set by the Cochrane Collaboration (Tables 1, 2, 3, 4, and 5). In case of multiple clinical studies in which the same restorations were analyzed at different time intervals, leading to different publications, the study with the longest follow-up was selected for definitive analysis.

### 2.3. Inclusion Criteria

**Figure 1:**
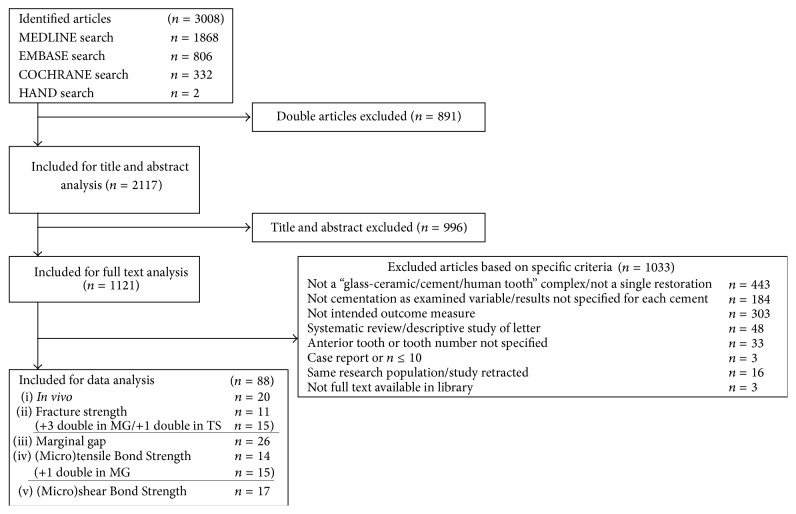
Algorithm of study selection procedure.

Only articles about glass-ceramic materials were considered. Clinically, the focus was on single-unit posterior restorations. Included studies should compare different cementation regimes and involve a “glass-ceramic/cement/human tooth” complex. Studies regarding the benefits of IDS attracted special attention. Descriptive studies (e.g., technical notes), systematic reviews, case reports, or studies with less than ten patients were excluded (Figure 1). Descriptions such as “selective double-bond technique,” “resin coating technique,” or “adhesive resin liner” were considered synonymous for IDS.

### 2.4. Data Extraction

The included studies were divided into* in vitro* and* in vivo* studies. For* in vitro* studies the data were organized according to the following topics: (micro)shear and (micro)tensile bond strength, fracture strength, and finally marginal gap and integrity. For* in vivo* studies survival and quality of survival were considered.

## 3. Results

The searches of MEDLINE (Pubmed), CENTRAL (Cochrane Central Register of Controlled Trials), and EMBASE resulted in 3008 publications. After exclusion of double publications, 2117 publications remained for title and abstract analysis. 1121 articles were hereafter included for full text analysis. Only a limited additional number of publications were found after checking the references of the included studies. Application of specified exclusion criteria resulted in 88 publications that could be included in the review. The exclusion criteria are described in Figure 1.

Interobserver agreement (Cohen's Kappa) regarding final inclusion or exclusion of studies that were proposed after full text analysis was 0.80 (IBM SPSS 22), which is generally considered to be a strong level of agreement [14]. Initial disagreements were generally caused by ambiguities in the study design or the characterization of materials used.

The included studies were assessed for their risk of bias according to the Cochrane library (Tables 1, 2, 3, 4, and 5). Assessment of allocation concealment and blinding of participants, personnel, and outcome assessors for included* in vitro* studies proved difficult and hardly ever applicable. Sequence generation and incomplete outcome data for* in vitro *studies are not explained in most cases but just named. Assessment “unclear” on incomplete outcome data generally implies that no missing data were reported. Most studies in this review did not report sequence generation; for* in vitro* studies the relevance of this can be subject of debate. For* in vivo studies* sequence generation, allocation concealment, and blinding were often assessed as “unclear,” because studies often did not describe these procedures. Overall the included studies had a low risk of bias. More specifically, a low risk of bias was assessed for shear bond strength studies, tensile strength studies, and marginal gap studies. An unclear risk of bias was assessed for fracture strength studies and* in vivo* studies.

**Figure 2:**
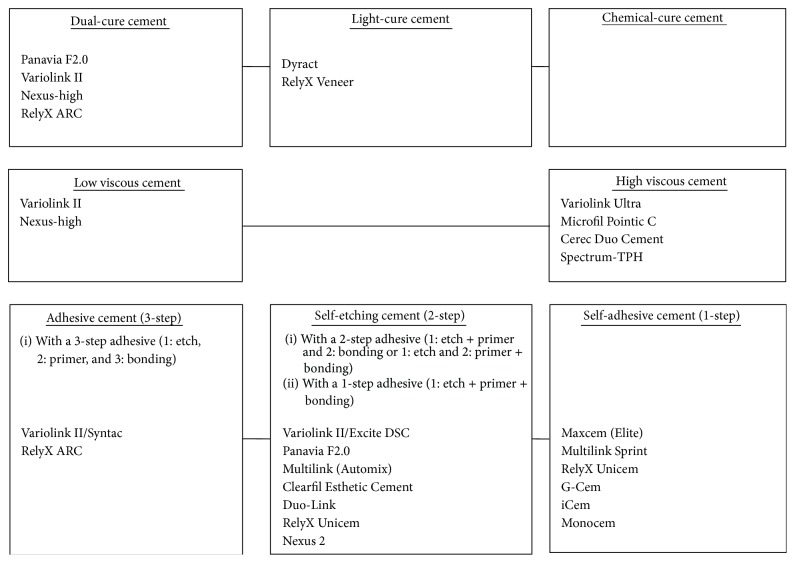
Choices in commonly used resin composite cements.

Because of their great variety it is important to divide contemporary resin cements into subgroups regarding their curing type, their viscosity, and whether they are either adhesive (with a 3-step adhesive), self-etching (with a 2-step or 1-step adhesive), or self-adhesive. This terminology is not used consistently in literature. An overview is presented in Figure 2. Cements that are named in this study will be specified as one of these three types, which usually depends on the adhesive used. Cement and adhesive system brand names, manufacturers, city, and countries of origin are presented in Table 6. Generally, different cement brands, cement types, or cementation techniques were compared in the included studies (e.g., water based cements among which are zinc phosphate (Harvard); polycarboxylate cement (Harvard); glass ionomer (Fuji I; Ketac-Cem; Dyract-Cem) and resin cements (Panavia 2; RelyX Unicem; Multilink; Maxcem; G-Cem; Prodigy; Nexus; Vita Cerec Duo Cement and Clearfil Esthetic cement)) in combination with several brands of glass-ceramic restorations. An overview of contemporary resin cements is presented in Figure 2.

### 3.1. *In Vitro* Studies

#### 3.1.1. (Micro)shear Bond Strength (*n* = 17 Studies)

Seventeen studies could be identified that met the inclusion criteria; their risk of bias is overviewed in Table 1.

In only one study different groups of luting agents were used and the authors concluded that zinc phosphate cement and glass ionomer cements produced the lowest shear bond strengths, whereas the highest shear bond strengths were found with two self-etching cements (Panavia F2.0 and Multilink) and one self-adhesive resin cement (RelyX Unicem) [15].

Several studies (*n* = 7, [16–22]) compared different resin cements in a shear bond strength test. Adhesive cements produced significantly higher shear bond strength values to dentin [16, 17]. When comparing self-adhesive cements with self-etching cements, the self-etching cements showed the highest bond strengths to dentin [18]. To enamel a self-etching cement (Variolink II/Excite DSC) produced better results compared to another self-etching cement (Clearfil Esthetic cement/ED primer II) [19]. When different self-etch resin cements were compared, Duo-Link showed the highest bond strength, followed by Variolink II (with Excite DSC), and Nexus 2 showed the lowest [20]. To dentin and enamel the adhesive cement Variolink II and the self-etch cement Panavia F2.0 showed the highest shear bond strengths, with Variolink II reaching the highest values [21]. In another study a similar conclusion was reached, but with no difference between Panavia F2.0 and Variolink II [22].

Others, using a push-out test, concluded that an adhesive cement (Variolink II/Syntac) did not perform better than three self-adhesive cements [23].

To enamel three different self-etching resin cements with different setting modes (dual-cure, light-cure, and flow) were compared in a microshear bond strength test; no significant differences were seen [24].

Four studies [25–28] focused specifically on the presumed benefits of IDS compared to Delayed Dentin Sealing (DDS). In two studies different dentin adhesives acted as an IDS and the authors concluded that they did not alter the retentive strength of adhesively luted ceramic restorations using either of the tested bonding systems [25, 26]. Two other studies concluded that IDS using Clearfil SE Bond resulted in improved shear bond strength compared to DDS [27, 28].

The application of fluoride or triclosan based desensitizing agents prior to adhesive cementation did not influence the shear bond strength [29], nor did laser-etching of the dentin compared to a self-etch (Clearfil Esthetic) and an etch-and-rinse cementation procedure (Variolink II) [30]. Application of a silane coupling agent to the ceramic surface after etching with hydrofluoric acid increases the shear bond strength [31].

In summary, some evidence supports the use of adhesive cement with respect to the shear bond strength compared to self-adhesive and self-etch systems when luting all ceramic materials to human dentin. There is little evidence to support the assumption that IDS improves the shear bond strength especially when Clearfil SE Bond was used.

#### 3.1.2. (Micro)tensile Bond Strength (*n* = 15 Studies)

Fifteen articles could be included investigating the effect of different cements on glass-ceramic restorative materials with a (micro)tensile bond strength test; their risk of bias is overviewed in Table 2.

When comparing different cement groups, glass ionomer cement (Aquacem) yielded far lower tensile bonding strengths (2-3 times) compared to a self-etch resin cement (Dicor LAC) [32].

In studies comparing different resin cements results were opposite or similar about which cement, self-etching or self-adhesive, resulted in the highest tensile bond strength [33–35] or obtained similar results for each cement, be it adhesive, self-etching, or self-adhesive [36]. Values were still worse than those obtained using adhesive luting agents [37] (personal communication) and [38]. But in another study this was contradicted because the self-etching cement did better than the adhesive cement [39]. When a less commonly used self-etching adhesive system (Super-Bond C&B) was used, a higher tensile bond strength was obtained compared to two other self-etching cements [40].

It was hypothesized that the tensile bonding strength is not so much dependent on the type of adhesive approach but more so on the chemical composition and viscosity of the cement used. Interestingly, the use of self-etch adhesive combined with a restorative composite (Clearfil SE Bond with Clearfil APX) yielded higher tensile bond stresses to dentin than dedicated self-adhesive, self-etch, and adhesive cements [39]. But no such difference was found when the same material (Clearfil APX) was used with another bonding system (Linerbond 2V) [41].

Overall, autocure leads to a lower microtensile bond strength when compared to dual-cure cement modes [42, 43]. Precuring of the adhesive layer increased tensile bond strengths [43].

As before, tensile bond strengths were also higher for enamel than for dentin, that is, in a study by Habekost et al. [44].

The effect of IDS on microtensile bond strength was tested in two studies. An IDS layer (one or two resin coatings) applied directly after preparation yielded higher values compared to applying it just prior to cementation or not at all. No temporary restorations were made [45, 46].

In summary, no one particular cement or adhesive system, be it self-etching, self-adhesive, or adhesive, showed overall superior results with respect to (micro)tensile bond strength. IDS improved microtensile bond strength in both included studies.

#### 3.1.3. Fracture Strength (*n* = 15 Studies)

Fifteen studies could be identified that met the inclusion criteria; their risk of bias is overviewed in Table 3. Seven studies [47–53] examined the effect of different cement groups like zinc phosphate, glass ionomer, or resin cements. Regardless of the preparation type, specimens with crowns that were adhesively cemented were stronger upon occlusal loading than those with conventionally cemented crowns [47]. Several other researchers came to a similar conclusion: zinc phosphate cements were associated with the lowest fracture loads [48] and adhesive cements increased fracture load significantly compared to glass ionomer and zinc phosphate cement [49, 50]. When comparing two self-adhesive cements with an adhesive cement and a glass ionomer cement, the self-adhesive cement (RelyX Unicem) revealed the highest fracture strength [51]. In one study the authors concluded that the cement type had no statistical significant effect on fracture resistance within the ceramic system [52] and in another study there were no differences found in fracture strength between glass ionomer, zinc phosphate, and composite resin cements [53].

Seven studies [44, 54–59] were included that examined the performance of different resins cements. Different variations of dentin bonding agents and resin luting materials were tested ((1) Mirage ABC and Mirage FLC; (2) Metabond; (3) All-bond 2 and Duo-Link; (4) Scotchbond multipurpose and 3M indirect porcelain bonding kit; (5) Mirage ABC and 3M indirect porcelain bonding kit). Mirage porcelain crowns were luted to premolars. The last two groups produced higher fracture strengths than the other three, suggesting that 3M indirect bonding kit was of significant influence [54]. In a study comparing two different dual-cure resin cements, it was unclear which adhesive system was used for each cement so the cements cannot be considered adhesive, self-etching, or self-adhesive. The authors hypothesize that cements with a higher flexural modulus exhibit higher values of fracture resistance for the ceramic/tooth assembly [55]. Others also suggest that the modulus of elasticity or the preparation design may be of larger influence than the adhesiveness of resin cements [44, 56]. In one study the authors concluded that the cement type had a significant effect on fatigue resistance in favor of the self-etching Panavia F2.0 [57], but other authors concluded Panavia F did the poorest, compared to other dual-cured resin cements [58]. When comparing a dual-cure cement (RelyX ARC) with a light-cure cement (RelyX Veneer), no significant differences in loads at failure among the tested cement group [59] were seen.

One study described the effect of the thickness of IDS materials (Clearfil SE Bond and Protect Liner F) on the fracture strength of IPS Empress II crowns cemented with Panavia F. The film thickness formed by Clearfil SE Bond and Protect Liner F increased the fracture load of IPS Empress II crowns [60].

In summary, teeth that are restored with an indirect glass-ceramic restoration, with respect to* in vitro* fracture strength of posterior adhesively cemented specimen, exhibit higher fracture strength with adhesive cements. Literature is inconclusive about the type of resin cement used. The modulus of elasticity is considered more important than the type of resin cement. There are no data found in the literature on fracture strength using contemporary glass-ceramics, such as lithium disilicate. So extrapolation of the findings to current materials and cementation protocols should only be done with great reservations. Little evidence supports the use of IDS in increasing the fracture load [60].

#### 3.1.4. Marginal Gap and Marginal Integrity (*n* = 26 Studies)

Twenty-six studies could be identified that met the inclusion criteria; their risk of bias is overviewed in Table 4. The effect of different viscosities was given special attention by several authors. The* in vitro* studies focusing on marginal gap and marginal integrity are too numerous to allow for individual discussion. Therefore the relevant findings evolving from these studies are outlined below.

A consistent finding is that the least microleakage and the best marginal adaptation are obtained when using a resin cement [50, 61–64]. These cements are also the least affected by artificial ageing. A glass ionomer cement exhibited a considerable drop in marginal adaptation after thermocycling, and such a finding seems relevant to clinical practice [51].

Four studies [65–68] focused on the effect of resin cements with different viscosities on marginal adaptation when luting a glass-ceramic restoration. The degree of viscosity was generally referred to as “high” (e.g., Variolink Ultra; Microfil Pontic C; Cerec Duo cement; Spectrum-TPH) or “low” (e.g., Variolink II; Nexus-high), without further physical description of the terms “high” or “low.” Both the initial size of the gap and the viscous properties of the luting agent were found to influence the final marginal (and also internal) gap width and marginal integrity. For relatively small discrepancies between the outline of the preparation and the margin of the restoration, low and high viscous cements result in similar interface widths after cementation [65]. Highly viscous cement is recommended for restorations with a larger luting space [66, 67]. Even luting spaces greater than 100 *μ*m can be partially compensated by a resin cement. In such cases highly viscous, filled composite cements are recommended when considering the quality of postcementation marginal integrity [68].

When applying resin cements, the degree of microleakage is generally higher on dentin margins than on enamel margins [57, 69–75]. Cement systems involving an etch-and-rinse approach result in higher percentages of gap-free margins in enamel than other luting systems, although in one study no difference is found between the etch-and-rinse cement (Panavia F2.0) and a self-adhesive resin cement (RelyX Unicem) [76]. However, self-etch adhesives and self-etch cements are also capable of sealing dentin tubules [77–79] or were even considered superior to the etch-and-rinse approach regarding this aspect [80].

In a study involving the cementation of partial crowns, preparation design was of no influence with respect to the size of the marginal gap [63].

Five studies [46, 75, 80–82] investigated the potential benefit of an IDS on the marginal gap. A temporary restoration was provided in only one of the studies [80]. In two studies the flowable composite extended to the cervical margin [75, 81], whereas in the other studies contamination of the margin with resin material was avoided [80, 82], which seems a relevant difference when looking at marginal adaptation. In most studies, less microleakage was seen when applying IDS compared to no IDS [75, 80–82]. However, one study found little difference in reducing microleakage at the dentin interface and even increased it at the enamel interface [46].

In summary, adhesive resin cements showed the least microleakage and are least affected by artificial aging. With a large marginal gap a highly viscous cement is recommended, when the gap is smaller there is no advantage but also no disadvantage of using a highly viscous cement. “Small” and “Large” are not further specified. Compared to enamel, there was generally more microleakage in dentin. There was little proof that with etch-and-rinse systems a higher percentage of gap-free margins could be obtained in enamel, compared to dentin. With self-etching systems and self-adhesive systems equivalent or even more gap-free margins were reached in dentin. IDS was generally considered of merit in reducing microleakage.

### 3.2. *In Vivo* Studies (*n* = 20 Studies)

There were twenty clinical studies on glass-ceramic restorations comparing different cementation protocols, but protocols and materials were seldom similar among different studies. Their risk of bias is overviewed in Table 5. Clinical performance is described as survival or success, often with additional qualitative measures such as USHPS criteria (United States Public Health Services criteria) and CDA-criteria (California Dental Association criteria).

Mirage fired feldspathic restorations were luted with either a dual-cure composite (Mirage) or a glass ionomer luting cement (Fuji I), resulting in 2% and 15% lost or fractured restorations, respectively, after a maximum observation period of 3 years. The predominant complication was adhesive bond failure at the cement-porcelain interface [83] as also concluded by others [84]. Clinically, good marginal adaptation and marginal seal and consequently little marginal discoloration, as well as good wear resistance, were observed, as expressed according to the USHPS criteria. No difference was seen in the cementation procedure. Marginal breakdown of this type of restoration cement with glass ionomer was also seen in a different study [85].

In another similar study restorations could be evaluated after 6 years with 12% and 26% failures, respectively. The difference was already obvious at the 3-year recall period [86]. In contrast to the former study, a deterioration of qualitative parameters was seen during the initial 3 years when judged according to USPHS-criteria regarding marginal adaptation and surface roughness for the dual-cure cement group and even more so for the glass ionomer group. The use of a light-cured (Mirage) instead of a dual-cured adhesive cement (Mirage FLC) presumably caused incomplete curing of the cement because of insufficient penetration of the light through the inlays, with concomitant reduction in fracture strength [87]. The insufficient penetration was associated with 80% versus 20% fracture of the Mirage restorations after a mean observation period of just over one year, especially in thin restorations (<2 mm). These restorations were so thin because a lining cement was used in case of deep preparations (Dycal or a glass ionomer). A similar protocol to protect the vital pulp was adopted in the study by van Dijken et al. [86], which should be kept in mind when extrapolating the results to other situations or current cementation protocols.

In another split mouth study, Cerec (Vita Mark II) inlays were cemented with either a dual-cured (Vita Cerec Duo Cement, Vita) or chemically cured resin cement (Cavex Clearfil F2) and evaluated according to the criteria of the California Dental Association. Twenty-three percent of the restorations were replaced, all from the dual-cured resin cement group within a 10-year period. Possibly, the self-curing capacity of the dual-cured resin cement was insufficient to achieve adequate hardening in order to withstand the stresses and strains that can arise in posterior regions. Although no differences in qualitative parameters were reported between baseline and the period after 10 years, acceptable scores for marginal discoloration after 10 years were seen more frequently in the dual-cured than in the chemically cured cement group (58% versus 78%) [88].

Klink and colleagues also used Vitablocs Mark II full crowns, partial crowns, and inlays luted with either Variolink II or RelyX Unicem. According to the CDA-criteria inlays and partial crowns performed well. Prevalence of complications or failure was highest for crowns. They concluded that success was related to patient factors and restoration type, not luting protocol [89]. Others also found that resin cement type had no influence on success using the same ceramic material [90]. It is noteworthy that the margins were entirely in enamel.

In a study by Gemalmaz and colleagues two adhesive cements (Variolink Ultra and Enforce) and a glass ionomer cement (Geristore) were used to lute Ducere LFC ceramic inlays resulting in 13%, 13%, and 33% failures, respectively, after a little more than 2 years. Margins were evaluated by SEM on gypsum models. Deterioration of marginal adaptation, rate of submargination, and marginal discoloration of surviving restorations luted with the glass ionomer cement were markedly inferior to those luted with the other two cements, with the restorations cemented with Variolink Ultra performing the best [91].

In a prospective dual-center study, the clinical behavior of adhesively luted pressed glass-ceramic restorations (Cergogold) was evaluated using two cementation regimens (personal communication). One group of restorations was luted with Definite Multibond primer with corresponding adhesive and definite cement and the other with Syntac classic (3-step) with Variolink Ultra cement. Survival rates were 93% and 95%, respectively, after 4 years, with the first group exhibiting more hypersensitivity shortly after cementation of the restoration (27% versus 0%). Hence both luting protocols provided similar results when compared according to USPHS criteria and by SEM [92]. A similar conclusion was reached in a different study by the same group involving other patients after 4 years of clinical service [93]. Two operators luted Cergogold inlays in 39 patients using protocols same as those previously described. Considerable interoperator differences were observed with respect to annual failure rate (0.6 versus 6.2%).

Lithium disilicate restorations were cemented with either a commercially available self-etching dual-curing cement (control, Multilink Automix) or a self-adhesive dual-curing “experimental” cement originating from the same company (experimental). Both cements had qualitatively similar results after 2 years of function as assessed by the modified USPHS criteria. All restorations functioned for 2 years without crown fracture or surface chipping. The undisclosed nature of the experimental cement leaves little room for practical comparison or interpretation. The publication did not mention the type of restoration that was provided (full, circumferential, or partial) [94]. For this restoration type, inlays luted with resin-modified glass ionomer cement (Fuji Plus F) or a self-cured resin composite cement (Panavia 21) yielded similar results after 5 years [95]. IPS Empress (leucite reinforced glass-ceramic) restorations were cemented with different adhesive approaches and can function successfully for 15 years [96]. Others also saw good long term results but described a significant amount of deterioration of marginal adaptation in the long run, even though modern adhesive procedures were used. Overall failure rates of this type of restoration were in the order of 8–10% after 10 years [97–99]. A classic etch-and-rinse approach (Syntac classic/Variolink II) produced better marginal integrity when cementing leucite reinforced glass-ceramic inlays than a contemporary self-adhesive resin cement (Relyx Unicem) after 2 years in function [100]. Another author favored dual-cure cements based on 12-year results [101], whereas the viscosity of the cement (low versus high) had no influence on success in a large prospective study after 10 years [102].

In conclusion, most included, rather heterogeneous clinical studies involve relatively old, no longer available restoration types or systems. The use of lining cements in several older protocols challenges external validity. Cementation protocols involving glass ionomer cements generally (but not always) result in more fracture and loss of restorations as well as poorer qualitative performance of surviving restorations compared to protocols involving adhesive resin cements. Studies comparing cementation protocols for more contemporary restorative materials (lithium disilicate) are rare and involve self-etching, self-adhesive, or adhesive procedures. None of these cementation protocols can be considered clearly superior in clinical performance on the basis of the reviewed literature.

There is limited evidence that light-cured resin cements perform worse than dual-cured cements, whereas solely chemically cured resin cements perform the best. Results obtained with technically challenging adhesive cementation procedures may be operator-dependent. Marginal deterioration is frequently reported, also when using adhesive cements.

No clinical studies evaluated the potential benefits of IDS protocols that were identified.

## 4. Discussion

This review is aimed at organizing knowledge regarding the cementation of glass-ceramic restorations, particularly posterior, single-unit ones, with a special emphasis on the possible merits of IDS. The topic is of interest to the clinician because of the growing number of all ceramic restorations that are being placed. They substitute metal and metal-ceramic crowns and are advantageous because they are relatively cheap in light of the current gold price and their manufacturing price and because of their superior esthetics. In early years, glass-ceramics were cemented with conventional cements like glass ionomers, with limited adhesive properties. This reflects on the results, as demonstrated in this comprehensive review, and consequently challenges the external validity of data subtracted from these studies to contemporary, strengthened glass-ceramics (leucite reinforced glass-ceramic and lithium disilicate). By removing superficial glass content by etching, glass-ceramics can be cemented adhesively and as a result allow nonretentive preparation forms, maintaining sound tooth tissue. This may help in avoiding endodontic complications.

Bonding to dentin has traditionally been considered to be more challenging than to enamel. IDS may provide better results with respect to the bonding capacity and it is possibly also more friendly to the pulp.

Over 3000 studies were initially identified for this review, but many were discarded, predominantly because they did not compare different cementation protocols or evaluated a “glass-ceramic/cement/human tooth” complex. The selection on articles in the English language only may have introduced some bias.

The* in vitro* and* in vivo* studies that were included proved dramatically heterogeneous. Consequently, they do not allow meta-analysis or relevant grouping because of different test methods (e.g., tooth and substrate preparation, dimension and geometry of the restoration or tested ceramic, tooth number, storage conditions, artificial aging/thermocycling or not, cyclic loading or not, cementation protocols (e.g., a single or a double adhesive layer), testing machines, standardization of the test method, crosshead speed of the testing device and the size of the steel ball during instrumentation, the use of a “stress breaker” such as a rubber dam, film thickness of luting cements, or (lack of) definition of outcome parameters, particularly the mode of failure). It was decided to include studies only if they compared cements or cementation procedures, thus correcting for the heterogeneity in some manner. Often it was complicated to categorize the cementation procedures into “adhesive,” “self-etching,” or “self-adhesive” because of the chosen bonding agents and the confusing way that they were applied and described.

With respect to the application of IDS, terminology and the clinical application in the literature regarding this procedure are different. The present authors regard IDS as a procedure in which a resin layer is applied immediately after preparation, followed by impression taking and the provision of a temporary restoration in combination with a temporary cement. Eventually, this restoration is replaced by a glass-ceramic one, which is luted to the reactivated IDS layer and the uncovered tooth structure by means of a resin cement. In the current review, when no temporary restoration was provided in an evaluated study, it is referred to as a “resin coating,” which is fundamentally different. The manner in which such an intermediate layer is applied and conditioned is also expected to be of influence and often different among studies that were included.

Nevertheless and possibly as a result of the rather rigorous inclusion and exclusion criteria, the included studies in the review are generally considered of good methodological quality as evaluated by Cochrane's collaboration tool of bias.

*In vitro* studies identify some differences in outcome resulting from the tested protocols or variables. These are generally not reflected in rather more crude, clinical outcome measures, such as survival of a restoration, presented in* in vitro* studies. Therefore it is tentatively suggested that when luting modern glass-ceramics to posterior teeth, adhesive protocols that are the most operator and patient friendly may be preferred.

## 5. Conclusion

Bearing in mind the shortcomings and limitations of this review as described above, the following conclusions are drawn.

From* in vitro* studies it can be concluded that adhesive systems (3-step, etch-and-rinse) show the best (micro)shear bond strength values compared to self-adhesive and self-etch systems when luting to human dentin. For (micro)tensile strength values or evaluation of the marginal gap no such preference can be identified on the basis of the reviewed literature. The highest fracture strength is obtained using adhesive cements, rather than water based cements like glass ionomer.

Clinical studies comparing cementation protocols for contemporary restorative glass-ceramic materials (lithium disilicate) are rare and involve self-etching, self-adhesive, and adhesive procedures. No marked clinical preference for one specific procedure could be demonstrated on the basis of the reviewed literature.

Few studies focus on the possible merits of IDS. The benefits are most convincingly illustrated by the favorable microtensile bond strengths when compared to negative or positive controls* in vitro*. No clinical trials have been performed and deleterious clinical consequences, be it objective or subjective, were not reported.

## Figures and Tables

**Table 1:** Assessment of risk of bias of included *in vitro* ((micro)shear bond strength) studies (*n* = 17) according to Cochrane collaboration's tool.

Adequate sequence generation?	Allocation concealment?	Blinding?	Incomplete outcome data addressed?	Free of selective reporting?	Free of other bias?	References
Unclear	NA	NA	Unclear	Yes	Yes	[22]
Unclear	NA	NA	No	Yes	Yes	[16]
Unclear	NA	NA	Yes	Yes	Yes	[27]
Unclear	NA	NA	Yes	No	Yes	[25]
Unclear	NA	NA	Yes	Yes	Yes	[26]
Unclear	NA	NA	Unclear	Yes	Yes	[29]
Unclear	NA	NA	Unclear	Yes	Yes	[28]
Unclear	NA	NA	Unclear	Yes	Yes	[23]
Unclear	NA	NA	Unclear	Yes	Yes	[30]
Unclear	NA	NA	Unclear	Yes	Yes	[31]
Unclear	NA	NA	Yes	Yes	Yes	[24]
Unclear	NA	NA	No	Yes	Yes	[21]
Unclear	NA	NA	Unclear	Yes	Yes	[20]
Unclear	NA	NA	Unclear	Yes	Yes	[15]
No	NA	NA	Yes	Yes	Yes	[19]
Unclear	NA	NA	Yes	Yes	Yes	[17]
Unclear	NA	NA	Yes	Yes	Yes	[18]

**Table 2:** Assessment of risk of bias of included *in vitro* ((micro)tensile bond strength) studies (*n* = 14) according to Cochrane collaboration's tool.

Adequate sequence generation?	Allocation concealment?	Blinding?	Incomplete outcome data addressed?	Free of selective reporting?	Free of other bias?	References

Unclear	NA	NA	Unclear	Yes	Yes	[34]
Unclear	NA	NA	Yes	Yes	No	[37]
Unclear	NA	NA	Yes	Yes	Yes	[33]
Unclear	NA	NA	Unclear	Yes	Yes	[44]
Unclear	NA	NA	Yes	Yes	Yes	[42]
Unclear	NA	NA	Unclear	Yes	Yes	[40]
Unclear	NA	NA	Unclear	Yes	Yes	[32]
Unclear	NA	NA	Yes	Yes	Yes	[35]
Unclear	NA	NA	Unclear	Yes	Yes	[45]
Unclear	NA	NA	Unclear	Yes	Yes	[43]
Unclear	NA	NA	Yes	Yes	Yes	[38]
Unclear	NA	NA	Yes	Yes	Yes	[39]
Unclear	NA	NA	Yes	Yes	Yes	[36]
Unclear	NA	NA	Unclear	No	Yes	[41]

**Table 3:** Assessment of risk of bias of included *in vitro* (fracture strength) studies (*n* = 11) according to Cochrane collaboration's tool.

Adequate sequence generation?	Allocation concealment?	Blinding?	Incomplete outcome data addressed?	Free of selective reporting?	Free of other bias?	References
Unclear	NA	NA	Unclear	No	Yes	[52]
Unclear	NA	NA	Unclear	Yes	Yes	[49]
Unclear	NA	NA	Unclear	Yes	Yes	[47]
No	NA	NA	Unclear	No	Yes	[48]
No	NA	NA	Unclear	No	Yes	[54]
Unclear	NA	NA	Unclear	Yes	Yes	[55]
Unclear	NA	NA	No	Yes	No	[59]
Unclear	NA	NA	Unclear	No	No	[53]
Unclear	NA	NA	Unclear	Yes	Yes	[58]
Unclear	NA	NA	Unclear	Yes	Yes	[56]
Unclear	NA	NA	Unclear	Yes	Yes	[60]

**Table 4:** Assessment of risk of bias of included *in vitro *(marginal gap) studies (*n* = 26) according to Cochrane collaboration's tool.

Adequate sequence generation?	Allocation concealment?	Blinding?	Incomplete outcome data addressed?	Free of selective reporting?	Free of other bias?	References
No	NA	NA	Unclear	Yes	Yes	[72]
Unclear	NA	NA	Unclear	Yes	Yes	[76]
Unclear	NA	NA	Unclear	No	Yes	[50]
Unclear	NA	NA	Unclear	Yes	Yes	[79]
Unclear	NA	NA	Unclear	Yes	Yes	[74]
Unclear	NA	NA	Unclear	Yes	Yes	[73]
Unclear	NA	NA	Yes	Yes	Yes	[71]
Unclear	NA	NA	Unclear	Yes	Yes	[63]
Unclear	NA	NA	Yes	Yes	Yes	[78]
Unclear	NA	NA	Unclear	No	Yes	[77]
Unclear	NA	NA	No	Yes	Yes	[70]
Unclear	NA	NA	Unclear	No	Yes	[62]
Unclear	NA	NA	Unclear	Yes	Yes	[66]
Unclear	NA	NA	Unclear	No	Yes	[67]
Unclear	NA	NA	Unclear	Yes	Yes	[80]
Unclear	NA	NA	Yes	Unclear	Yes	[75]
Unclear	NA	NA	Yes	Unclear	Yes	[57]
Unclear	NA	NA	Yes	Yes	Yes	[82]
Unclear	NA	NA	No	Yes	Yes	[46]
Unclear	NA	NA	Unclear	No	Yes	[65]
Unclear	NA	NA	Yes	No	Yes	[61]
Unclear	NA	NA	Unclear	Yes	Yes	[51]
Unclear	NA	NA	Unclear	No	Yes	[64]
Unclear	NA	NA	Unclear	Yes	Yes	[81]
Unclear	NA	NA	Yes	No	Yes	[68]
Unclear	NA	NA	Yes	Yes	Yes	[69]

**Table 5:** Assessment of risk of bias of included *in vivo* studies (*n* = 20) according to Cochrane collaboration's tool.

Adequate sequence generation?	Allocation concealment?	Blinding?	Incomplete outcome data addressed?	Free of selective reporting?	Free of other bias?	References
Unclear	Unclear	Unclear	Yes	Yes	Yes	[83]
Unclear	Unclear	Unclear	Yes	No	Yes	[99]
No	Unclear	Unclear	No	No	No	[94]
Unclear	Unclear	Unclear	Yes	Yes	Yes	[93]
Unclear	Unclear	Unclear	Yes	No	Yes	[101]
Unclear	Unclear	Unclear	Yes	Yes	Yes	[91]
Unclear	Unclear	Unclear	Yes	No	No	[87]
Unclear	Unclear	Unclear	Yes	No	No	[89]
Unclear	Unclear	Unclear	Yes	Yes	Yes	[97]
Unclear	Unclear	Unclear	Yes	Yes	Yes	[98]
Unclear	Unclear	Unclear	Yes	Yes	Yes	[92]
Unclear	Unclear	Unclear	Yes	Yes	Yes	[84]
Unclear	Unclear	Unclear	Yes	No	Yes	[88]
Unclear	Unclear	Unclear	Yes	No	Yes	[102]
Yes	Unclear	Yes	Yes	Yes	Yes	[100]
Unclear	Unclear	Unclear	Unclear	No	No	[95]
Unclear	Unclear	Unclear	Yes	No	Yes	[96]
Unclear	Unclear	Unclear	Yes	Yes	No	[86]
Unclear	Yes	Unclear	Yes	Yes	Yes	[85]
Unclear	Yes	Unclear	Yes	Yes	Yes	[90]

**Table 6:** Cement and adhesive system brand names, manufacturers, city, and countries of origin.

Cement and adhesive system brand names	Manufacturers	City	Countries of origin
Adapter SingleBond 2	3M ESPE	Seefeld	Germany
All-bond 2	Bisco Inc.	Schaumburg, IL	USA
Authentic	Ceranay	Stuttgart	Germany
Aquacem	Dentsply deTrey	Konstanz	Germany
Biomer	Dentsply Caulk	Milford, DE	USA
Cavex Clearfil F2	Cavex	Norden	Germany
Cergo	DeguDent	Hanau	Germany
Cergogold	DeguDent	Hanau	Germany
Chemiace II	Sun Medical	Moriyama City	Japan
Clearfil Esthetic Cement	Kuraray	Tokyo	Japan
Clearfil Protect Bond	Kuraray	Tokyo	Japan
Clearfil SA	Kuraray	Tokyo	Japan
DeTrey Zinc	Dentsply deTrey	Konstanz	Germany
Definite Multibond primer	DeguDent	Hanau	Germany
Definite cement	DeguDent	Hanau	Germany
Dicor cement	Dentsply	York, PA	USA
Dicor LAC	Dentsply deTrey	Konstanz	Germany
Ducere LFC	Ducere	Rosbach	Germany
Duo-Link	Bisco Inc.	Schaumburg, IL	USA
Dycal	Dentsply Caulk	Milford, DE	USA
Dyract-Cem	Dentsply DeTrey	Konstanz	Germany
ED primer II	Kuraray	Tokyo	Japan
Enforce	Dentsply	São Paulo	Brazil
Excite (DSC)	Ivoclar Vivadent	Schaan	Liechtenstein
Finesse	Dentsply Ceramco	Burlington, NJ	USA
Fleck's	Mizzy Inc.	Cherry Hill	USA
Fuji I	GC Corp.	Tokyo	Japan
Fuji Plus (F)	GC Corp.	Tokyo	Japan
G-Cem	GC Corp.	Tokyo	Japan
Geristore	Dent-Mat	Santa Maria	USA
GC Fuji Cem	GC Corp.	Tokyo	Japan
Go!	3M ESPE	Seefeld	Germany
Harvard	Richter-Hoffman	Berlin	Germany
Harvard cement	Harvard Dental	Berlin	Germany
iCem	Heraeus Kulzer	Hanau	Germany
Illusion Universal Cementation System	Bisco Dental Products	Richmond, BC	Canada
IPS E.max Press	Ivoclar Vivadent	Schaan	Liechtenstein
IPS Empress (I) (II)	Ivoclar Vivadent	Schaan	Liechtenstein
Ketac-Cem	3M ESPE	St. Paul, MN	USA
Linerbond 2V	Kuraray	Osaka	Japan
Metabond	Sun Medical	Moriyama City	Japan
Maxcem	Kerr-Hawe	Orange, CA	USA
Microfil Pontic C	Heraeus Kulzer	Hanau	Germany
Mirage	Chameleon Dental	Kansas City, KA	USA
Mirage ABC	Chameleon Dental	Kansas City, KA	USA
Mirage FLC	Chameleon Dental	Kansas City, KA	USA
Multilink (Automix)	Ivoclar Vivadent	Schaan	Liechtenstein
Multilink primer	Ivoclar Vivadent	Schaan	Liechtenstein
Multilink Sprint	Ivoclar Vivadent	Schaan	Liechtenstein
Nexus	Kerr Corp.	Orange, CA	USA
Nexus 2	Kerr Corp.	Orange, CA	USA
Nexus 3	Kerr Corp.	Orange, CA	USA
Nexus-high	Kerr Corp.	Orange, CA	USA
Noritake Super porcelain	Noritake Dental Supply Co., Ltd.	Nagoya	Japan
One Coat Bond	Coltene/Whaledent AG	Altstätten	Switzerland
Optibond FL	Kerr Corporation	Orange	United States
Panavia 21	Kuraray	Osaka	Japan
Panavia F2.0	Kuraray	Osaka	Japan
Panavia F	Kuraray	Osaka	Japan
Protect Liner F	Kuraray	Osaka	Japan
Prodigy	Kerr Corp.	Orange, CA	USA
RelyX ARC	3M ESPE	St. Paul, MN	USA
RelyX Veneer	3M ESPE	St. Paul, MN	USA
RelyX Unicem (Clicker)	3M ESPE	St. Paul, MN	USA
Single Bond	3M ESPE	Seefeld	Germany
Self-etching primer A+B	Ivoclar Vivadent	Schaan	Liechtenstein
SmartCEem 2	Dentsply Caulk	Milford, DE	USA
Spectrum-TPH	Dentsply Caulk	PA	USA
SpeedCEM	Ivoclar Vivadent AG	Schaan	Liechtenstein
Super-Bond C&B	Sun Medical	Moriyama City	Japan
Super porcelain EX-3	Noritake Kizai Co.	Nagoya	Japan
Syntac (classic)	Ivoclar Vivadent	Schaan	Liechtenstein
Temp Bond	Kerr	Corporation, Orange	United States
Tetric flow	Ivoclar Vivadent	Schaan	Liechtenstein
Universal glass ionomer	Super Dent	Westbury, NY	USA
Variolink II	Ivoclar Vivadent	Schaan	Liechtenstein
Variolink II base	Ivoclar Vivadent	Schaan	Liechtenstein
Variolink II refill	Ivoclar Vivadent	Schaan	Liechtenstein
Variolink II Ultra	Ivoclar Vivadent	Schaan	Liechtenstein
Vitadur Alpha	Vita	Bad Säckingen	Germany
Vita Cerec Duo Cement	Coltene/Whaledent AG	Altstätten	Switzerland
Vita Mark II	Vita	Bad Säckingen	Germany
